# Rates of clinically apparent heparin-induced thrombocytopenia for unfractionated heparin vs. low molecular weight heparin in non-surgical patients are low and similar

**DOI:** 10.1186/1477-9560-3-4

**Published:** 2005-04-04

**Authors:** Charles FS Locke, John Dooley, Jonathan Gerber

**Affiliations:** 1 Johns Hopkins Community Physicians Department of Internal Medicine 2360 W. Joppa Rd., Suite 306 Lutherville, MD 21093 USA

## Abstract

With the growing use of low-molecular-weight heparins (LMWH) for the treatment and prevention of venous thromboembolism (VTE), it is important to provide an evidence-based comparison with unfractionated heparin (UFH) concerning rates of heparin-induced thrombocytopenia (HIT). Such comparisons are essential in clinical decision-making and cost-modeling. In this paper we review data regarding non-surgical (medical) patients. We conclude that the lack of uniform evaluation and standardized testing for HIT in the current literature precludes making a reliable estimate of the relative risk of HIT in UFH vs. LMWH in either the treatment or prevention of VTE in non-surgical patients. However, current data suggest that the risk of thrombocytopenia and HIT is low and similar for non-surgical patients who receive either LMWH or UFH.

Heparin-induced thrombocytopenia (HIT) is recognized as a rare, but potentially devastating complication of heparin therapy because of its association with arterial and venous thrombosis [1]. HIT is mediated by antibodies which recognize an antigen formed by the binding of platelet factor 4 to heparin [2]. The now widespread use of low-molecular-weight heparins for a variety of indications previously reserved exclusively for unfractionated heparin has generated interest in comparing the relative rates of HIT between the two classes of heparin.

In 1995 Warkentin examined rates of HIT in patients undergoing elective hip arthroplasty who had been randomized to receive either unfractionated heparin (UFH) or low molecular weight heparin (LMWH) for thromboprophylaxis [3]. Warkentin reported that HIT occurred in 9 of 332 patients who received UFH and in none of 333 patients who received LMWH (2.7 percent vs. 0 percent). In addition, development of heparin-dependent IgG antibodies and thrombotic events associated with thrombocytopenia were more common in patients treated with UFH than in those treated with LMWH.

Recently, using different criteria for HIT (an absolute drop in platelet count of 50% or greater vs. platelet count less than 150,000 cells /ml), Warkentin reanalyzed these same data and found the difference in the observed rate of HIT was even more pronounced, 8 times greater (4.8% vs. 0.6%) in UFH compared to LMWH for prophylaxis of venous thromboembolism (VTE) in patients undergoing elective hip arthroplasty [4].

Warkentin's results are supported by a recent study by Walenga et al which carefully evaluated sera from three clinical studies [5]. Walenga found that LMWH was less likely to generate H-PF4 antibodies than UFH and less likely to result in clinical HIT. Walenga also noted that LMWH were more likely to generate IgA and IgM antibodies rather than IgG antibodies, which are associated with clinical HIT. However, the sera reviewed by Walenga all were from orthopedic surgical patients.

Authors in the medical literature often generalize Warkentin's results, applying them to medical as well as surgical patients [6-8]. Further, it has been suggested that differences in rates of HIT represent an advantage of LMWH over UFH in VTE prophylaxis in non-surgical patients [7,9].

However, we do not think the Warkentin data can be applied with confidence to non-surgical patient populations. In non-surgical (medical) patients, the rate of HIT with UFH is reported to be much lower than in Warkentin's analysis of surgical patients. For example, an earlier study cited in Warkentin's 1995 paper reported an incidence of HIT of only 0.3% for non-surgical patients who received therapeutic intravenous UFH [10].

To evaluate the relative rates of HIT in non-surgical patients we reviewed recent studies that compared UFH to LMWH in either the treatment or prevention of VTE in medical (non-surgical) patients. We chose studies available to us through PubMed. In our review, we found 11 trials that reported either HIT (which, as Warkentin points out, does not have a uniform definition), thrombocytopenia, "severe thrombocytopenia" or some combination of the above. Our findings are listed in the table 1.

**Table 1:** Treatment duration and reported adverse event rates in studies comparing UFH vs. LMWH heparin in the treatment and prophylaxis of VTE

**Treatment of VTE**							
**Study**	**Treatment duration (days)**		**Reported Adverse Event**	**UFH**		**LMWH**	

	**UFH**	**LMWH**		**%**	**n/N**	**%**	**n/N**

Merli [11]	≥ 5^‡^	≥ 5^‡^	T*	1.4	4/290	2.0	12/610
Koopman [12]	6.1	6.5	T(u)	2.5	5/198	1.5	3/202
Levine [13]	5.5	5.8	T	1.9	3/253	2.0	5/247
			T w/o exp.	0.4	1/253	0.4	1/247
Hull [14]	not stated		T(u)	1.0	1/103	3.1	3/97
Columbus Invest [15]	5.8	6.3	HIT	0.3	1/308	0.0	0/304
Simmoneau [16]	7.0	7.3	HIT	0.3	1/308	0.0	0/304
**Prophylaxis of VTE**							

**Study**	Treatment duration (days)		**Reported Adverse Event**	**UFH**		**LMWH**	

	**UFH**	**LMWH**		**%**	**n/N**	**%**	**n/N**

Harenberg, 1990 [17]	10	10	**		-/82		-/84
Harenberg, 1996 [18]	10	10	T(d)	0.5	4/780	0.0	0/810
PRIME [19]	7	7	T (n)	0.0	0/482	0.0	0/477
Bergmann [20]	10	10	HIT†	0.4	1/223	0.0	0/216
PRINCE [21]	10	10	T		NR		NR

Key:

T:Thrombocytopenia, defined as platelet count less than 100,000 cells/ml,

*One case of "immune thrombocytopenia reported in this study". Case was in the LMWH group but had received UFH prior to randomization.

T(u) – Thrombocytopenia-undefined in study

T w/o exp. – Thrombocytopenia with "no apparent explanation"

NR: not reported,

**: "Thrombocyte count did not change in either group".

T(d): A decrease in platelet count (values ranging between 40,000 and 80,000/microliter) was observed in four patients with UF and in none with LMW heparin. No severe thrombocytopenia was observed.

T(n): "There was no decrease in platelet count due to enoxaparin or Ca-heparin."

† One patient with drop in platelet count from 149 K cells/ml to 87 K during treatment. Platelet count rose to 280 K post study. No sequallae from thrombocytopenia.

^‡ ^Average length of treatment not stated. Treatment length "at least five days".

The studies cited in the tables are heterogeneous in the endpoints used. Given the variability in definition of thrombocytopenia among the trials and the lack of standardized and routine evaluation for HIT in any of the above studies we feel it is currently impossible to estimate the relative risk of HIT in UFH vs. LMWH in either the treatment of VTE or prevention of VTE in non-surgical patients. However, the data do suggest that thrombocytopenia is rather uncommon with either heparin therapy. Insofar as the rate of HIT must be less than that of thrombocytopenia, HIT is likely to be an infrequent event as well.

A recently published study provided a rigorous analysis of H-PF4 antibodies in patients treated for deep vein thrombosis with LMWH vs. UFH [22]. In this study, H-PF4 antibodies (measured by a commercial ELISA method) developed in 9.1% of patents in the UFH group vs. 2.8% of patients in the LMWH group (both treated for 5–7 days). However, there was only one occurrence of HIT with thrombosis among 356 patients in the UFH group vs. no occurrences of HIT among 374 patients in the LMWH group. This study, we feel, is consistent with both Warkentin's data regarding orthopedic surgery patients and the data presented in our table above; namely, LMWH induces H-PF4 antibodies at a lower rate than UFH but that clinical incidence of HIT in non-surgical patients is too small to statistically differentiate.

Unfortunately, the generalization to medical patients of Warkentin's data regarding HIT rates for orthopedic surgical patients persists in the literature. As recently as 2004, a meta-analysis comparing heparins for the treatment of pulmonary embolism cited "the lower risk for...heparin-induced thrombocytopenia" as an advantage of LMWH over UFH [7]. As justification, this paper references a review article [8] which in turn, references the 1995 Warkentin paper [3] which, as discussed above, involved exclusively orthopedic surgical patients.

One possible explanation as to why the studies of treatment and prevention of VTE in medical patients have not demonstrated a difference in thrombocytopenia or HIT rates is because the length of treatment in these studies may be too short for patients to develop HIT. In a study which clearly demonstrated a difference in HIT rates for LMWH vs. UFH following cardiopulmonary bypass surgery, 4 patients developed significant levels of Heparin-PF4 antibodies in days 3–5 postoperatively vs. 75 patients in days 7–10 post operatively [23]. Similarly, in Warkentin's data, thrombocytopenia typically developed 6–13 days after surgery (and of heparin therapy) and thrombotic events occurred 7–17 days after surgery [3].

In a recent study, Girolami et al reviewed 598 consecutive patients admitted to a medical ward with either a therapeutic or prophylactic indication for UFH [24]. HIT was not observed in any of the 238 patients who received UFH for a therapeutic indication. The authors speculate that HIT was not observed in these patients because duration of heparin was less than one week. There were 5 cases of HIT observed in the 598 patients (0.8%), all in those patients who received heparin for prophylactic indication. These cases occurred from day 8 to day 22 of therapy and the three observed associated thrombotic events occurred on days 10–21 of therapy. Such data are consistent with the College of American Pathologists (CAP) 2002 recommendations that platelet monitoring for HIT should focus on days 4–10 after starting heparin [25]. In addition, the CAP position on monitoring for HIT differentiates medical vs. surgical patients, with more frequent platelet count monitoring recommended for surgical patients. The CAP calls "postoperative" patients receiving UFH at "highest" risk for HIT, while "medical" patients receiving UFH are considered at "intermediate" risk [25].

The Girolami study further support the position that, in most cases, the use of heparin for the treatment of VTE is limited to the first 5–7 days of treatment and that heparin (either LMWH or UFH) is discontinued before clinical HIT, as evidenced by either thrombocytopenia and/or thrombosis, generally occurs. Similarly, the strong correspondence of length-of-treatment with the likelihood of development of H-PF4 antibodies and HIT is likely important in prophylaxis of non-surgical patients as well.

In the studies we reviewed, the length of heparin pharmacoprophylaxis was generally 7–10 days. Despite this length of treatment being ostensibly long enough for patients to develop laboratory-evident HIT we suggest that 7–10 days of therapy is too short of a duration for many cases of potential clinically-evident HIT to manifest. This could limit the potential clinical import of differences in rates of HIT for UFH and LMWH. Indeed, our experience is that length-of-stay in our institution for most of our medical patients eligible for pharmacoprophylaxis is 10 days or less. Additionally, in the studies of "medical patients" we reviewed, the risk low and similar risk of either thrombocytopenia, HIT or thrombotic complications of HIT in the UFH and LMWH groups may also be, in part, due to the absence of surgical activation of PF-4 in these patients.

Accurate assessment of the risks and benefits of competing therapies is paramount to sound cost-effective decision-making. At our institution, acquisition costs for branded LMWH are approximately 15 times that of generic UFH, a factor that would certainly favor the latter if efficacy and safety are similar. In Europe and Canada, where cost differences between LMWH and UFH are less pronounced, clinical decision-making and cost-modeling may be different than in the United States. Indeed, the latest Amercian College of Chest Physicians' (ACCP) guidelines on antithrombotic therapy recognize that "the cost for low-molecular-weight heparin (LMWH) is high in the United States, but low in most European countries. Thus, in instances in which small benefits accrue to patients from the use of LMWH in comparison to the use of unfractionated heparin, the choice in favor of LMWH may be clear in Europe, but much less clear in North America" [26]. However, given the serious (and expensive) nature of complications from HIT, true differences in clinical HIT with thrombosis between UFH and LMWH would affect significantly safety considerations as well as total health care cost-modeling between the two therapies. Unfortunately, we feel sufficient information in this area are currently lacking.

We encourage investigators to make a rigorous evaluation of HIT using new definitions proposed by Dr. Warkentin in his 2003 paper [4] as part of any future studies comparing LMWH and UFH for either the treatment or prevention of VTE in non-surgical patients to better define the risk of this important clinical problem.
